# Intravascular Lithotripsy-Assisted Transfemoral TAVI: The Copenhagen Experience and Literature Review

**DOI:** 10.3389/fcvm.2021.739750

**Published:** 2021-09-22

**Authors:** Fadi J. Sawaya, Vilhelmas Bajoras, Maarten Vanhaverbeke, Christina Wang, Gintautas Bieliauskas, Lars Søndergaard, Ole De Backer

**Affiliations:** ^1^The Heart Centre, Rigshospitalet, Copenhagen University Hospital, Copenhagen, Denmark; ^2^Department of Cardiology, American University of Beirut Medical Center, Beirut, Lebanon

**Keywords:** transcatheter aortic valve implantation (TAVI), access, transfemoral (TF), calcified, lithotripsy

## Abstract

Transcatheter aortic valve implantation (TAVI) is currently an established therapy for elderly patients with symptomatic severe aortic valve stenosis across all surgical risk categories. Access is an important aspect when planning for and performing TAVI. The superiority of a transfemoral (TF) approach compared to a transthoracic (transapical, direct aortic) approach has been demonstrated in several studies. Recently, the introduction of intravascular lithotripsy (IVL) has made it possible to treat patients with calcified iliofemoral disease by TF approach. This article aimed to provide a comprehensive overview on the following aspects: (1) preprocedural planning for IVL-assisted TF-TAVI; (2) procedural aspects in IVL-assisted TF-TAVI; (3) outcomes of IVL-assisted TF-TAVI in an experienced TAVI center; and (4) literature review and discussion of this new emerging approach.

## Introduction

Transcatheter aortic valve implantation (TAVI) is currently an established therapy for elderly patients with symptomatic severe aortic valve stenosis (AS) across all surgical risk categories (1, 2). Access is an important aspect when planning for and performing TAVI. The superiority of a transfemoral (TF) approach as compared to a transapical or direct aortic approach has been demonstrated in a meta-analysis of randomized controlled trials comparing TAVI and surgical aortic valve replacement (SAVR) (3). TF access should be the first choice for TAVI whenever the patient's anatomy allows this approach.

Iliofemoral arterial disease is not uncommon in TAVI candidates with advanced age and multiple medical co-morbidities. Improved insertion profile and flexibility of TAVI delivery systems has allowed to increase the percentage of TAVI procedures performed by TF approach.

**Figure 1:**
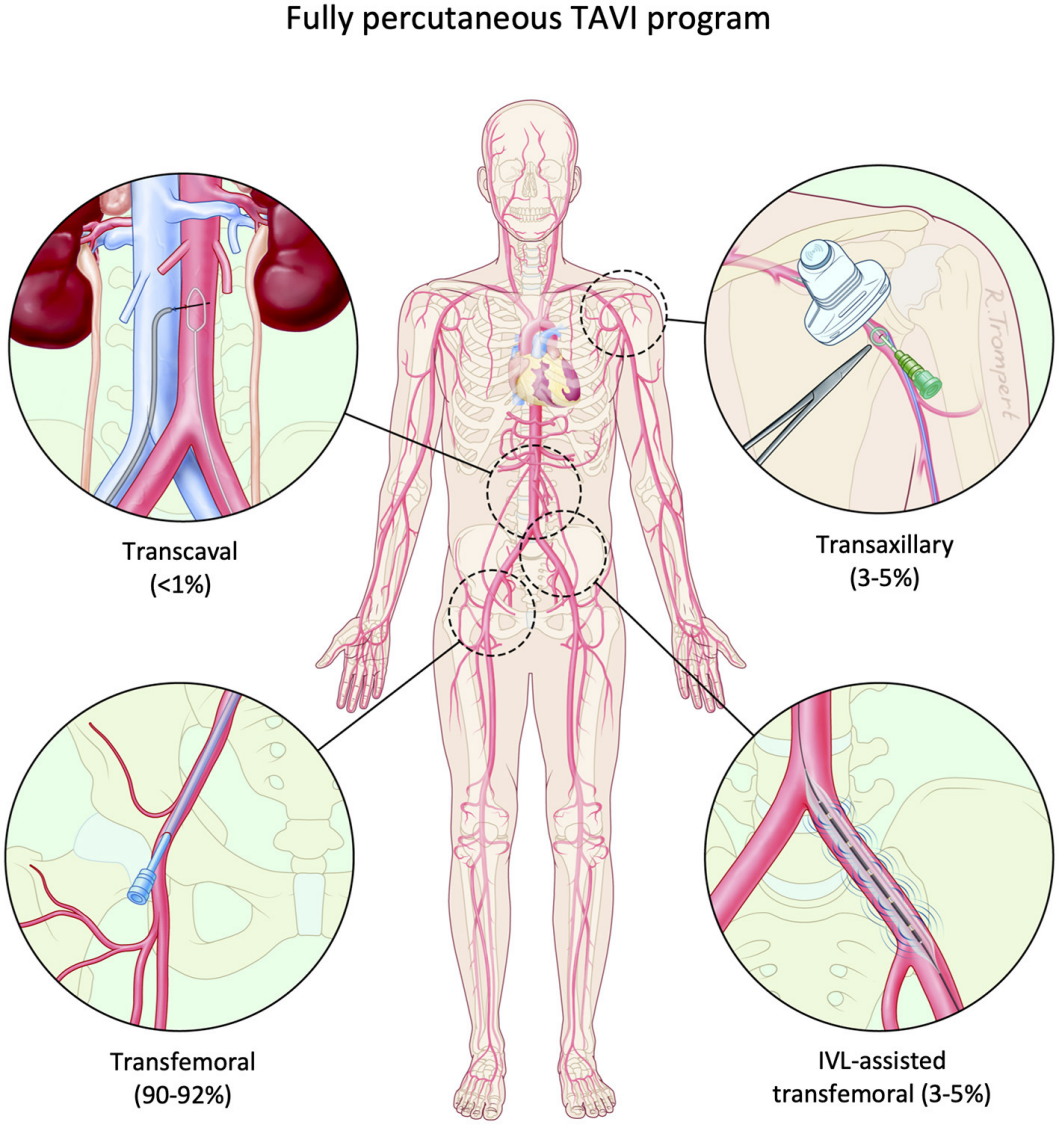
Fully percutaneous TAVI program. A fully percutaneous TAVI program is safe and feasible using different access routes: routine transfemoral, IVL-assisted transfemoral, percutaneous transaxillary and transcaval approaches. IVL, intravascular lithotripsy; TAVI, transcatheter aortic valve implantation.

If standard TF access is deemed unsuitable after meticulous computed tomography (CT) angiography analysis, second line alternative accesses including transaxillary, transsubclavian, transcarotid, transcaval and transapical approaches can be considered (Figure 1).

Recently, intravascular lithotripsy (IVL) technology–using circumferential pulse sonic pressure waves to modify both vessel intimal and medial calcifications–has emerged as a potential treatment option for calcified, stenotic iliofemoral artery disease (4). By controlled fracturing of the arterial calcifications by means of acoustic waves, the vascular compliance is modified and percutaneous transluminal angioplasty (PTA) and/or passage with a TAVI delivery system can be performed in a safe and efficient manner. Peripheral IVL has been previously studied as a stand-alone treatment demonstrating excellent safety and efficacy (5) along with registry data on its use in TAVI patients with calcified iliofemoral disease (6). Consequently, IVL therapy can potentially expand the patient cohort eligible for TF-TAVI and minimize the patient cohort requiring alternative non-TF access.

This article aimed to provide a comprehensive overview on the following aspects: (1) preprocedural planning for IVL-assisted TF-TAVI; (2) procedural aspects in IVL-assisted TF-TAVI; (3) outcomes of IVL-assisted TF-TAVI in an experienced TAVI center; and (4) literature review and discussion of this new emerging approach.

## Preprocedural Planning for IVL-Assisted TF-TAVI

A thorough CT analysis is essential in the planning of IVL-assisted TF-TAVI. Due to its ability to accurately assess vessel architecture, a contrast CT angiogram provides greater predictive value for vascular complications when compared to traditional invasive angiography. Both iliofemoral arteries should be routinely analyzed from the infrarenal abdominal aorta to the femoral bifurcation. By using semi-automatic post-processing software, centerline placement and curved multiplanar reformats can be obtained. Manual verification of the centerline should always be performed to ensure accurate arterial tracking and appropriate intra-luminal location of the centerline (7). Attention should be paid to the risk for calcium blooming artifacts and appropriate windowing should be used to correct for this. It should also be ensured that all measurements are performed perpendicular to the long axis of the vessel.

**Figure 2:**
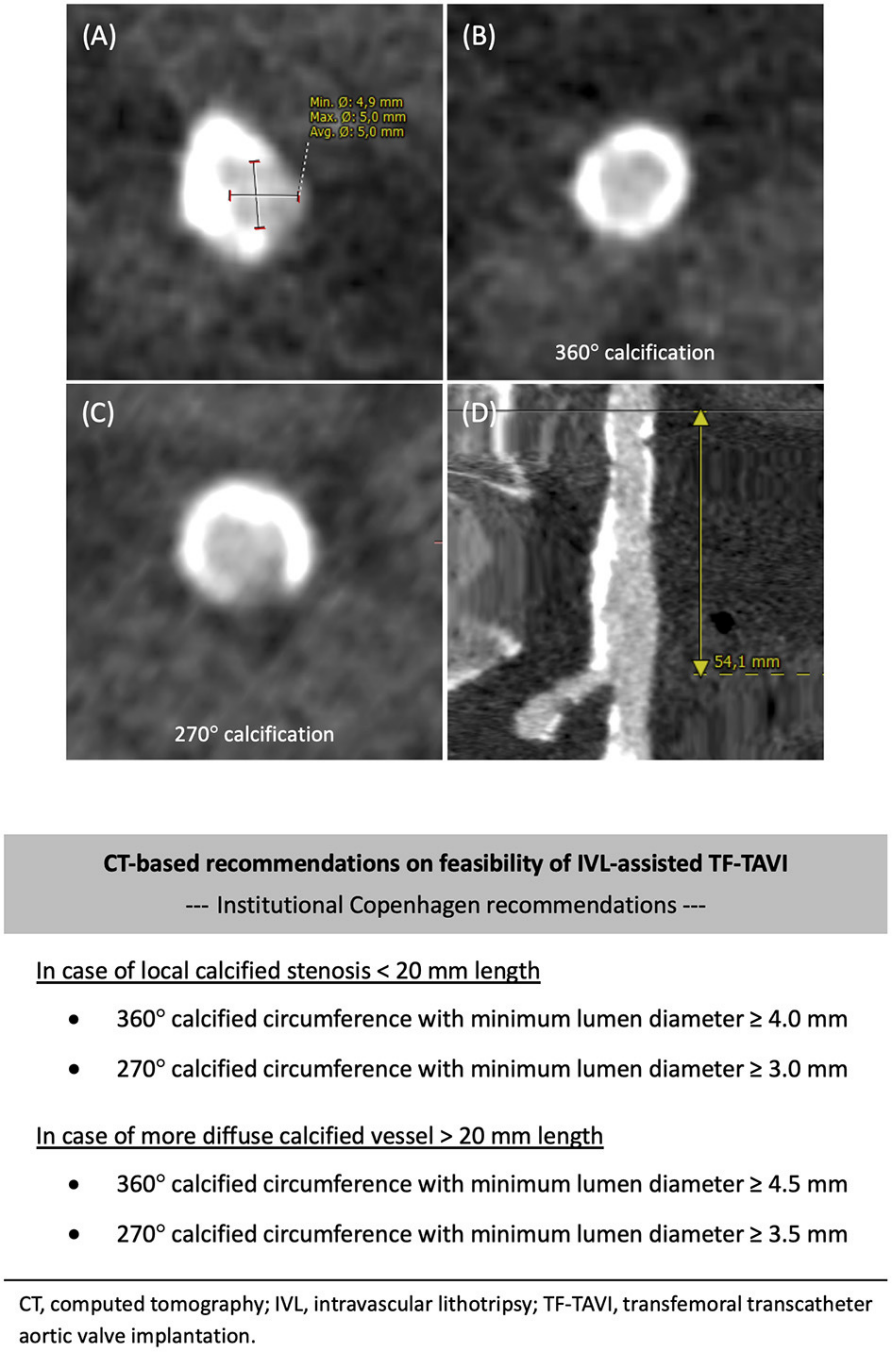
Preprocedural CT analysis. Preprocedural computed tomography (CT) angiography analysis of the iliofemoral access. Standard assessments include: **(A)** Maximal and minimal diameter measured at the minimal luminal diameter (MLD) of the vessel. **(B,C)** Circumference of calcification: 360° or horseshoe-like (270°) calcification. **(D)** Total length of the vessel calcification(s). Copenhagen recommendations on feasibility of IVL-assisted TF-TAVI based on CT angiography analysis. IVL, intravascular lithotripsy; TAVI, transcatheter aortic valve implantation; TF, transfemoral.

The following measurements should be routinely made and reported: the minimal luminal diameter (MLD), the maximal luminal diameter, the mean lumen diameter (maximal luminal diameter + minimal luminal diameter/2) (Figure 2A), the vessel area at the site of the most critical stenosis, and the degree of vessel tortuosity (8).

Calcifications along the vessel course are of particular significance. Arc (°) and morphology of the calcifications should be assessed; whether these are arranged in a circumferential (360°) or horseshoe-like (270°) pattern (Figures 2B,C), especially when borderline luminal diameters are present. Such calcifications will limit arterial expansion to accommodate large-bore introducer sheaths or TAVI delivery systems, potentially increasing the risk of dissection or perforation. Circumferential calcifications may not be appreciated at single-plane invasive angiography, underlining the added value of a contrast CT angiogram. Also, the length of the calcified segment(s) should be assessed (Figure 2D).

There is currently no consensus to indicate which candidates are deemed eligible for IVL-assisted TF-TAVI. The CT-based criteria as reported in Figure 2 have been utilized in our center as a reference to indicate whether IVL-assisted TF-TAVI is feasible, or not. These should be considered general recommendations–these need to be adapted to the clinical setting and available materials and expertise in each center. In general, the more diffuse in length and more circumferential in perimeter the calcifications are, the higher the threshold of MLD will be to be acceptable for IVL-assisted TF-TAVI, and vice versa.

An alternative, non-TF access should be considered if IVL-assisted TF-TAVI is deemed not feasible after Heart Team discussion. Therefore, assessment of alternative accesses should be routinely performed in TAVI candidates with significant calcified iliofemoral artery disease. Axillary and/or carotid arteries can be analysed using the same techniques and parameters as mentioned above. For axillary access, a left-sided approach is typically preferred in view of the aortic angulation. Also, the presence of an implantable electronic devices (pacemaker, defibrillator) in the left upper chest and/or a history of left internal mammary artery grafting should be noted.

If a transcaval approach is considered, the presence and size of a calcium-free window of the infrarenal aortic wall adjacent to the inferior vena cava should be assessed (9). The target entry site's distance below the renal arteries and above the aorto-iliac bifurcation and the level relative to the lumbar vertebrae should be measured. Any interposed structures (e.g., bowel) should also be noticed.

When planning for IVL-assisted TF-TAVI, extra attention should be paid to the anticipated puncture site at the common femoral artery (CFA). At CT analysis, a calcium-free window at the anterior wall of the CFA should be identified, such that an ideal arterial puncture and subsequent successful vascular closure can be secured. It is also recommended to make note of the anticipated location of the puncture site and its relation to the femoral bifurcation and the femur head. Additionally, CT analysis should include measuring the maximal luminal diameter at the intended puncture site; this to secure correct sizing of an endovascular balloon and/or covered or bare stent, if needed.

## Procedural Aspects of IVL-Assisted TF-TAVI

**Figure 3:**
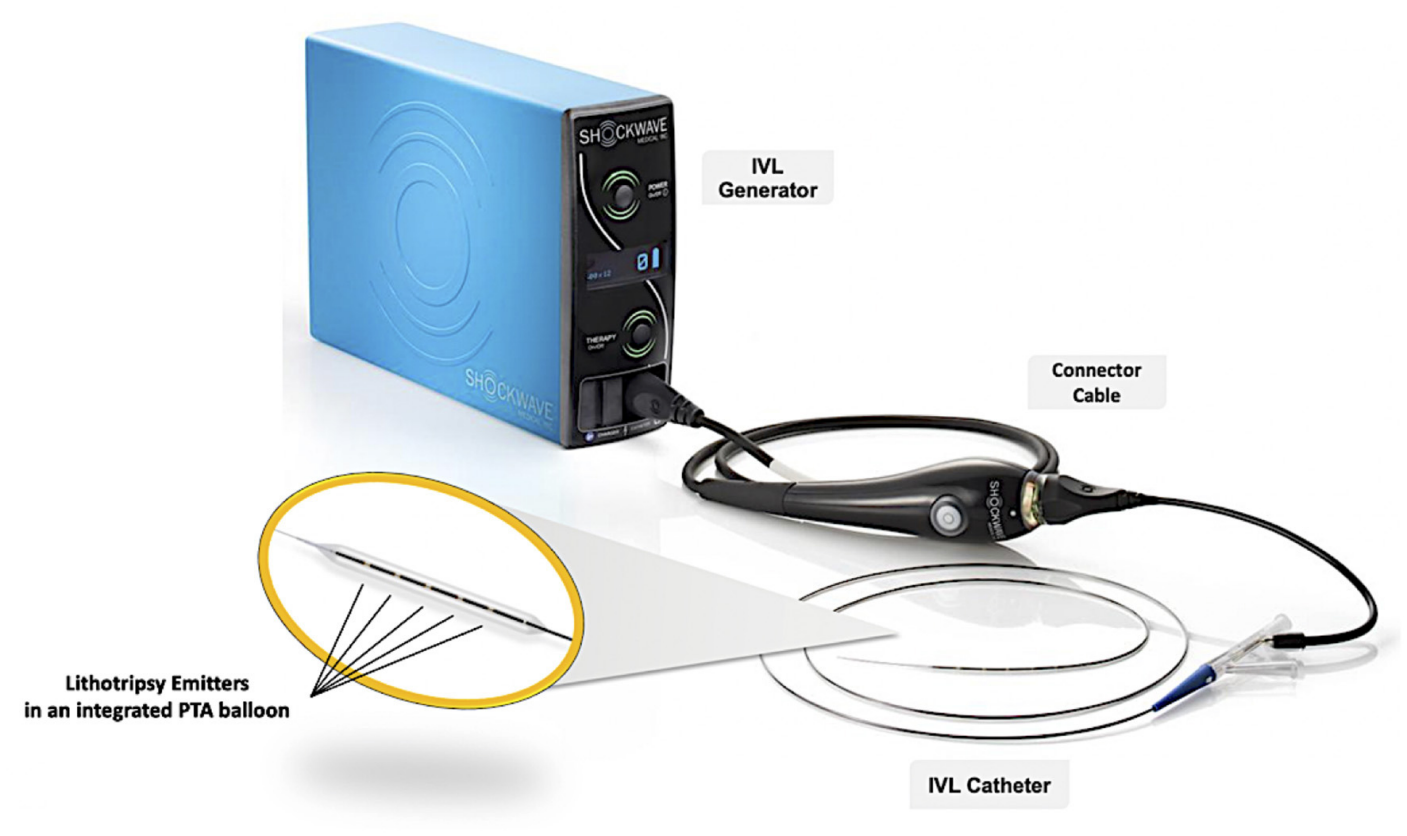
Shockwave IVL system components. The Shockwave IVL system consists of three components: (1) a portable IVL generator, (2) a connector cable with magnetic connection and push-button activation, and (3) an IVL catheter that houses an array of lithotripsy emitters enclosed in an integrated PTA balloon. IVL, intravascular lithotripsy; PTA, percutaneous transluminal angioplasty.

The Peripheral IVL Shockwave^TM^ catheter (Shockwave Medical Inc, CA, USA) is indicated for lithotripsy-enhanced low-pressure PTA of calcified, stenotic peripheral arteries, including the iliofemoral artery (4). The system consists of three parts: a generator, a connector cable, and an IVL catheter that houses an array of lithotripsy emitters enclosed in an integrated PTA balloon (Figure 3). The peripheral IVL catheter consists of an over-the-wire (OTW) balloon catheter which is compatible with any 0.014” guidewire. The balloons have a length of 60 mm and are available in multiple diameter sizes ranging from 3.5 to 7.0 mm in 0.5 mm increments. The generator produces 3 kV of energy that travels through the connector cable and catheter to the lithotripsy emitters at one pulse per second. To achieve balloon-vessel wall apposition, the integrated balloon is inflated to 4 atm by an in-deflator. A small electrical discharge at the emitters vaporizes the fluid and creates a rapidly expanding bubble within the balloon. The emitters positioned along the length of the balloon create a localized field effect within the vessel, with series of sonic waves passing through the vascular tissue and selectively fracturing the calcifications in the vessel architecture. Lithotripsy is administered in 30 pulses per cycle. After pulse emission, the integrated balloon is then further inflated to nominal pressure (6 atm) in order to maximize luminal gain. The cycle can be repeated, as needed, until satisfactory luminal diameter gain is obtained. The IVL catheter can be moved freely to other target lesions to deliver lithotripsy up to a total of 10 cycles (300 lithotripsy pulses in total). In case a long(er) segment of the iliofemoral artery needs IVL treatment, it is recommended to start balloon inflation and IVL treatment at the level of the iliac bifurcation and work its way down to the CFA. The positive effects of IVL are more pronounced as the vessel calcific burden is higher, especially in the presence of circumferential (360°) calcium by inducing multiple calcium fractures with significant increase of the luminal area.

An IVL-assisted TF-TAVI procedure can be performed in local anesthesia or under general anesthesia. The possible advantage of performing such TAVI procedures under general anesthesia is that ‘bail-out' alternative access can still be an option, if IVL-assisted TF-TAVI turns out not to be technically feasible.

**Figure 4:**
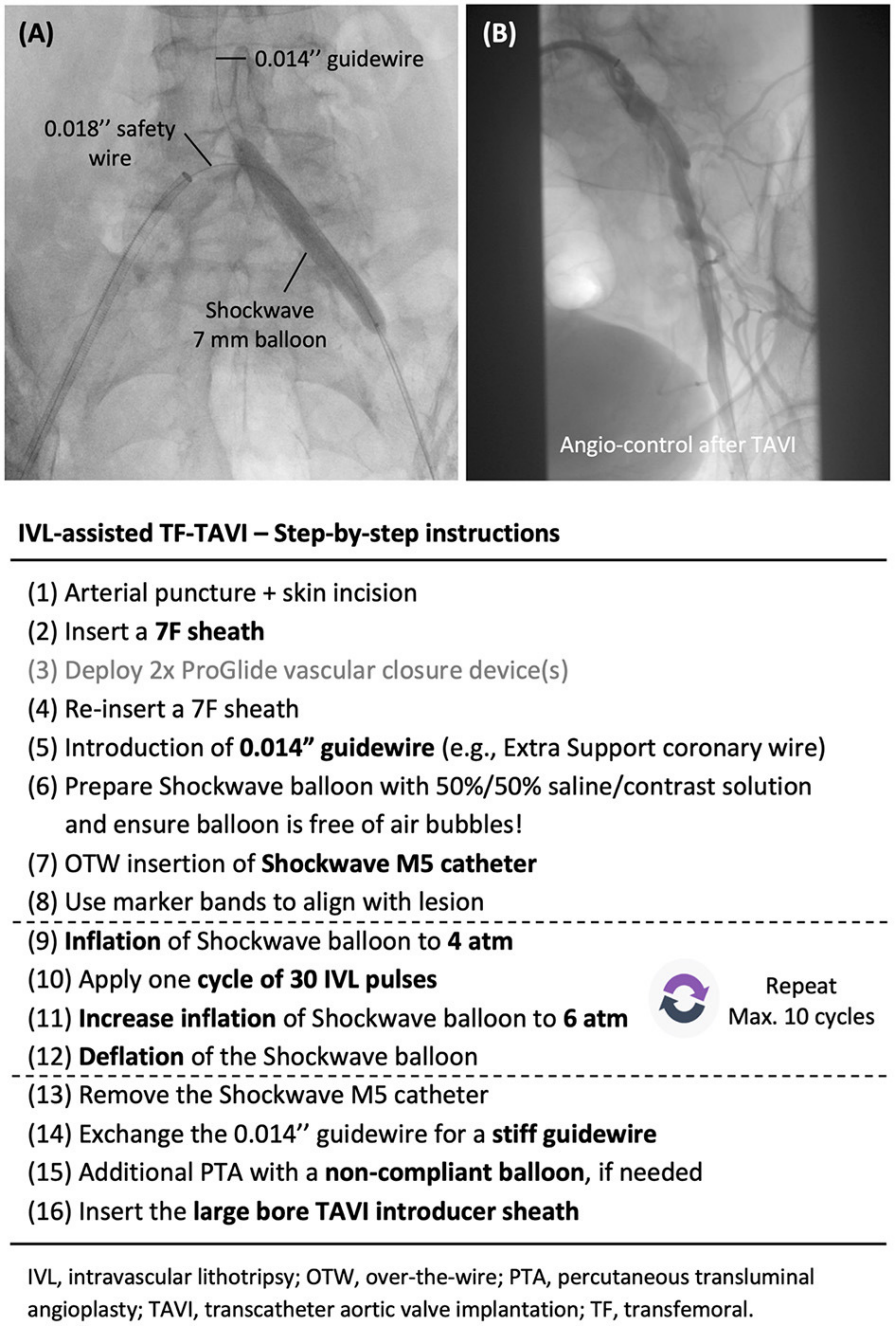
Step-by-step guide. **(A)** Treatment of a stenotic calcified common iliac artery with a 7 mm Shockwave IVL catheter. The use of a contralateral or ipsilateral 0.018” safety wire is strongly recommended. **(B)** Angiographic control with contrast injection is recommended after withdrawal of the large bore introducer sheath and vascular closure. IVL, intravascular lithotripsy; TAVI, transcatheter aortic valve implantation; TF, transfemoral.

A step-by-step guide on how to perform IVL-assisted TF-TAVI can be found in Figure 4. The use of a contralateral or ipsilateral 0.018” safety guidewire is strongly recommended, especially in case of TF-TAVI in an ‘hostile' iliofemoral setting (Figure 4A). This can secure a potential bailout procedure in case of complications during iliofemoral intervention, vascular closure device failure or unsuccessful closure at the puncture site.

Access to the CFA can be performed under fluoroscopic or ultrasound guidance. Fluoroscopy-guided puncture can clearly delineate the level of the puncture site in relation to the femoral head and femoral artery bifurcation, while ultrasound-guided puncture has the advantage of identifying a calcium-free spot and avoiding the use of contrast. The vascular closure strategy can be either suture- or collagen-based (e.g., ProGlide^TM^ or MANTA^TM^ vascular closure devices, respectively). A standard 7 Fr introducer sheath can accommodate all sizes of IVL catheter. After successfully crossing the target lesions with a 0.014” guidewire, the IVL catheter can be backloaded to the guidewire after careful preparation of the integrated balloon system. IVL therapy can be delivered to the target lesions as described above (Figure 4A). Post-IVL adjunctive PTA with a non-compliant balloon (e.g., Z-MED II balloon; Braun Medical Inc, USA) is not infrequently performed–this to further maximize the luminal expansion and gain. After adequate preparation of the vessel, the large-bore sheath can then be introduced over a stiff guidewire. Subsequent introduction of the TAVI device should be without any problem.

After closure of the large-bore vascular access, angiographic control of the IVL-treated iliofemoral artery and access site is recommended (Figure 4B). If vascular complications arise, bailout balloon tamponade or peripheral stenting can be performed at the iliac level and/or at the arteriotomy site. Based on a meticulous pre-procedural planning, this material should be available in the catheterization or hybrid lab in case of emergency.

## Real-World Outcomes in a Single Center Experience

Between November 2018 and August 2021, a total of 50 TAVI candidates with severe calcified iliofemoral disease were deemed unsuitable for standard TF-TAVI (*N* = 35) or at high risk for vascular complications in case standard TF-TAVI would be performed (*N* = 15). However, these patients were considered suitable and were accepted for IVL-assisted TF-TAVI at our center. All procedures were performed according to best clinical practice. The choice of transcatheter heart valve type/size and vascular closure device was at the operator's discretion, after careful evaluation of the patient's anatomical and clinical characteristics (10). Only self-expanding transcatheter aortic valves were used in this series, as balloon-expandable transcatheter heart valves have a larger profile, making these balloon-expandable valves not the best choice when treating patients with borderline calcified access vessels. The baseline characteristics of these patients are listed in Table 1.

**Table 1:** IVL-assisted transfemoral TAVI experience in Copenhagen.

**Patient characteristics**	***N* = 50**
Age	78.3 ± 6.7
Male	31 (62%)
Baseline left ventricular ejection fraction, %	51 ± 11
Aortic valve area, cm^2^	0.7 ± 0.2
Mean aortic gradient, mmHg	45 ± 11
STS surgical risk score, %	2.6 ± 1.5
Lesion characteristics and TAVI procedure details	
Target lesion location (*N* = 89 in total)	
Common iliac	41 (46%)
External iliac	30 (34%)
Common femoral	18 (20%)
Reference vessel diameter, mm	8.7 ± 2.2
Target lesion diameter, mm	4.9 ± 1.1
Vessel diameter stenosis, %	55 ± 13
Target lesion length, mm	37 ± 16
Maximal arc calcification, degrees	302 ± 69
Procedural details	
Use of contralateral/ipsilateral safety wire	39/11 (78/22%)
IVL catheter 7.0 mm x 60 mm size	50 (100%)
Post-IVL adjunctive PTA with non-compliant balloon	46 (92%)
Residual vessel stenosis, %	29 ± 10
TAVI performed at same time as IVL	50 (100%)
Successful transfemoral delivery of TAVI device	50 (100%)
Access outcomes	
Vascular complications related to IVL	
Vessel rupture/perforation	0 (0)
Dissection requiring bare metal stenting	1 (2%)
Vascular complications related to the puncture site	7 (14%)
Need for additional balloon inflation/AngioSeal	2 (4%)
Need for covered stent placement	5 (10%)
VARC-3 defined major vascular complications	0 (0)

*Values are mean ± SD or n (%). IVL, intravascular lithotripsy; PTA, percutaneous transluminal angioplasty; TAVI, transcatheter aortic valve implantation; VARC, Valve Academic Research Consortium*.

In all cases, TF delivery of the TAVI device (Portico, *N* = 22; Evolut, *N* = 18; Acurate, *N* = 10) was successful after IVL treatment and adjunctive PTA (in 92% of cases) of the iliofemoral axis. Mean reference vessel diameter was 8.7 mm, target lesion diameter 4.9 mm, with an average diameter stenosis of 55%. Average maximal arc calcium was 302°. All IVL treatments were performed with a 7 mm Shockwave^TM^ balloon catheter; this because the Shockwave^TM^ balloon is a compliant balloon which is inflated at low pressures and partial recoil of the IVL-treated vascular segment can be anticipated. No arterial perforation or rupture was noted. There was encountered one common iliac artery type A dissection requiring bare stent implantation. No other IVL-related vascular complications occurred. In 7 patients (14%), there was a (partial) closure device failure at the puncture site, requiring covered stent placement in 5 patients (10%) or a balloon inflation and/or AngioSeal insertion in two other patients. Importantly, these complications at the puncture site were not related to IVL treatment and, overall, there were no VARC-3 defined major vascular complications noted. Further details on the lesion characteristics and TAVI procedures are listed in Table 1.

This preliminary single center experience shows the safety and effectiveness of IVL vessel preparation to facilitate TF-TAVI in patients traditionally considered not suitable for the TF approach, thereby avoiding the usage of alternative access for TAVI.

## Literature Review and Discussion

IVL application for peripheral artery disease (PAD) treatment was first investigated in a pre-market European study, the single-arm DISRUPT PAD I study (4). Peripheral IVL was shown to result in a significant reduction in stenosis severity with high acute luminal gain and minimal vessel injury–this in 35 patients with severely calcified femoropopliteal disease. This study was followed by the DISRUPT PAD II trial, a non-randomized multi-center trial including 60 patients with complex calcified PAD, which confirmed that IVL was associated with large acute luminal gain, minimal complications, and minimal need for stenting (5). DISRUPT PAD III was a prospective, non-randomized, multi-center study conducted to assess the ‘real-world' acute safety and effectiveness of the Shockwave^TM^ peripheral IVL system, showing that peripheral IVL was safe and effective to treat symptomatic occlusive disease and could enable large-bore sheath advancement through calcified iliac arteries (11). Acute results included low residual stenosis with minimal complications, which were similar to those previously reported in the DISRUPT PAD I and II studies, and this despite a ‘real-world' population with a high burden of severe arterial calcifications.

Before the introduction and availability of peripheral IVL, hostile iliofemoral access remained a challenging scenario for TF-TAVI. Staniloae et al. reported on a TF-TAVI case series including 28 subjects with hostile iliofemoral access: twelve patients required preparatory PTA prior to advancing the TAVI device, one patient underwent orbital atherectomy followed by PTA prior to TAVI. Notably, two patients had a failed TAVI procedure because of common iliac artery perforation requiring stenting–both cases had severe circumferential calcifications (360°, luminal diameters 4.6 and 4.8 mm) and both perforations occurred while attempting to pass the device through the stenotic segment. Both patients underwent TAVI at a later date via an alternative approach (8). Another strategy to tackle challenging TF access has been the upfront placement of covered stents in the iliofemoral axis as “endoconduits” followed by PTA to larger diameters causing controlled rupture of the access vessels. This strategy is also known as the “pave and crack” technique. This technique is at the extreme end of endovascular vessel preparation and should not be routinely performed. Compared to this technique, peripheral IVL therapy offers a safe alternative option, preserving important side branches and avoiding stenting costs.

The use of IVL does not only reduce the risk of vascular complications in TF-TAVI, but also helps to maximize the use of the TF approach for TAVI. Preserving the TF access route and minimizing the use of alternative accesses should be strived for when planning TAVI, as only TF-TAVI has been shown to have superior clinical outcomes compared to SAVR. In a study by (12) it was reported that there was a marked reduction of the need for alternative access TAVI since the introduction of peripheral IVL in their practice. The use of the transapical approach fell from 13% in 2016 to 0.8% in 2018, while the use of the TF approach increased from 85% in 2016 to 94% in 2018. (6) reported the largest series of IVL-assisted TF-TAVI so far; in all 42 patients, a successful delivery of the TAVI device was achieved (6). There were no cases of iliofemoral arterial perforation or dissection requiring stent implantation. Studies on peripheral IVL-assisted large-bore access are summarized in Table 2 (13).

**Table 2:** Summary of studies on IVL-assisted large-bore transfemoral access.

	**Di Mario et al. (6) *N* = 42**	**Armstrong et al. (11) *N* = 17**	**Price et al. (13) *N* = 9**
Age	80.5 ± 7.3	72.5 ± 8.3	79.3 ± 9.8
Procedure type			
TAVI	42 (100%)	4 (24%)	4 (44.4%)
TEVAR	-	-	1 (11%)
EVAR	-	13 (76%)	1 (11%)
Fenestrated EVAR	-	-	3 (33%)
Reference vessel diameter, mm	8.1 ± 1.6	8.4 ± 2.5	N/A
Vessel diameter stenosis, %	59 ± 18	79 ± 19	N/A
Target lesion length, mm	37 ± 23	43 ± 22	42 ± 31
IVL catheter size			
5.0 x 60 mm	1 (2%)	N/A	0 (0)
6.0 x 60 mm	4 (10%)	N/A	4 (40%)
6.5 x 60 mm	4 (10%)	N/A	0 (0)
7.0 x 60 mm	33 (79%)	N/A	6 (60%)
Number of pulses per lesion	166 ± 68	234 ± 144	N/A
Successful TF delivery of TAVI device	42 (100%)	17 (100%)	9 (100%)
Complications			
Perforation	0 (0)	0 (0)	1 (11%)
Dissection requiring stenting	0 (0)	0 (0)	2 (22%)

*Values are % (n) or mean ± SD. N/A, not available. EVAR, endovascular aortic repair; IVL, intravascular lithotripsy; TAVI, transcatheter aortic valve implantation; TEVAR, thoracic endovascular aortic repair; TF, transfemoral*.

## Conclusions

Peripheral IVL appears to be a safe and effective solution for TAVI candidates with co-existing iliofemoral calcifications. Using peripheral IVL to facilitate TF access should be part of the TAVI algorithm, aiming to maintain the safety profile and superior outcomes of traditional TF-TAVI. More research is needed to improve the understanding on anatomical selection for IVL in TAVI candidates. Operators performing IVL-assisted TF-TAVI should be familiar with endovascular interventions and bailout solutions, not so much to treat IVL-treated lesions, but to be able to treat any vascular complication that may occur at the puncture site.

## Author Contributions

OD and FS: conception or design of the work. VB, CW, and OD: data collection, analysis, and interpretation. FS and OD: drafting the article. GB, LS, and OD: critical revision of the article. FS, VB, CW, GB, LS, and OD: final approval of the version to be published. All authors listed have made a substantial, direct and intellectual contribution to the work, and approved it for publication.

## Conflict of Interest

OD received speaker fees from Shockwave Medical Inc. The remaining authors declare that the research was conducted in the absence of any commercial or financial relationships that could be construed as a potential conflict of interest.

## Publisher's Note

All claims expressed in this article are solely those of the authors and do not necessarily represent those of their affiliated organizations, or those of the publisher, the editors and the reviewers. Any product that may be evaluated in this article, or claim that may be made by its manufacturer, is not guaranteed or endorsed by the publisher.

## Abbreviations

AS: aortic valve stenosis
CFA: common femoral artery
CT: computed tomography
IVL: intravascular lithotripsy
MLD: minimal luminal diameter
OTW: over-the-wire
PAD: peripheral artery disease
PTA: percutaneous transluminal angioplasty
SAVR: surgical aortic valve replacement
TAVI: transcatheter aortic valve implantation
TF: transfemoral.
